# Supplementary figures and images for: Insufficient yet improving involvement of the global south in top sustainability science publications
Source: PLoS One. 2022 Sep 1;17(9):e0273083. doi: 10.1371/journal.pone.0273083 (PMC9436092; doi:10.1371/journal.pone.0273083)

**S1 Figure:** Procedure followed for the literature survey

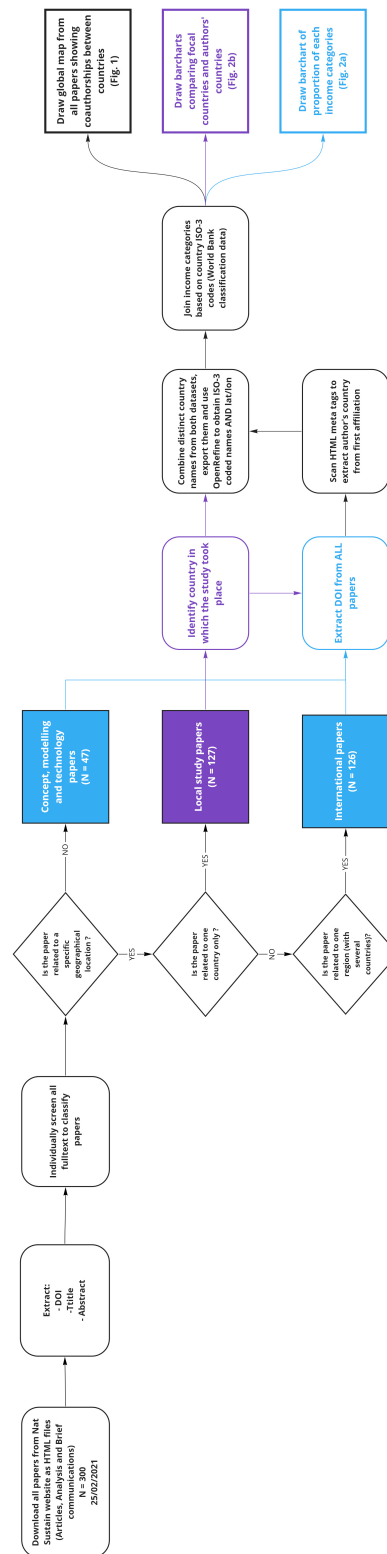

Supplement: S1 Fig — (PDF) [file pone.0273083.s001.pdf]

**S3 Figure:** Contribution of LLMIC authors in *Nature Sustainability* publications (2018-2021).

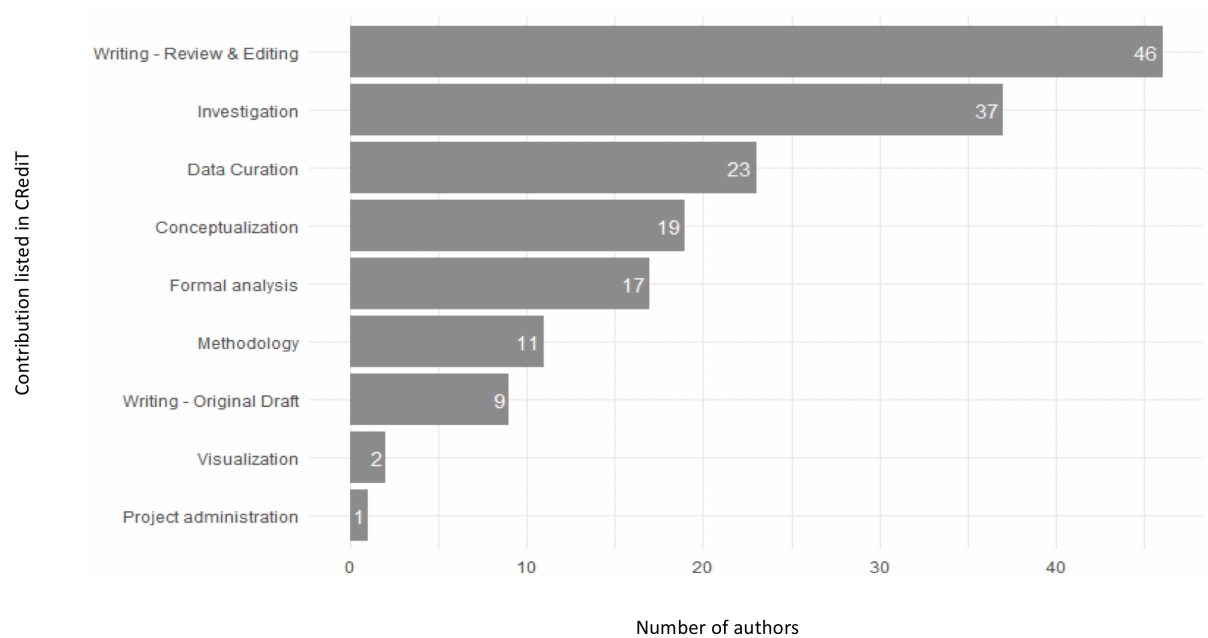

Supplement: S3 Fig — (PDF) [file pone.0273083.s003.pdf]

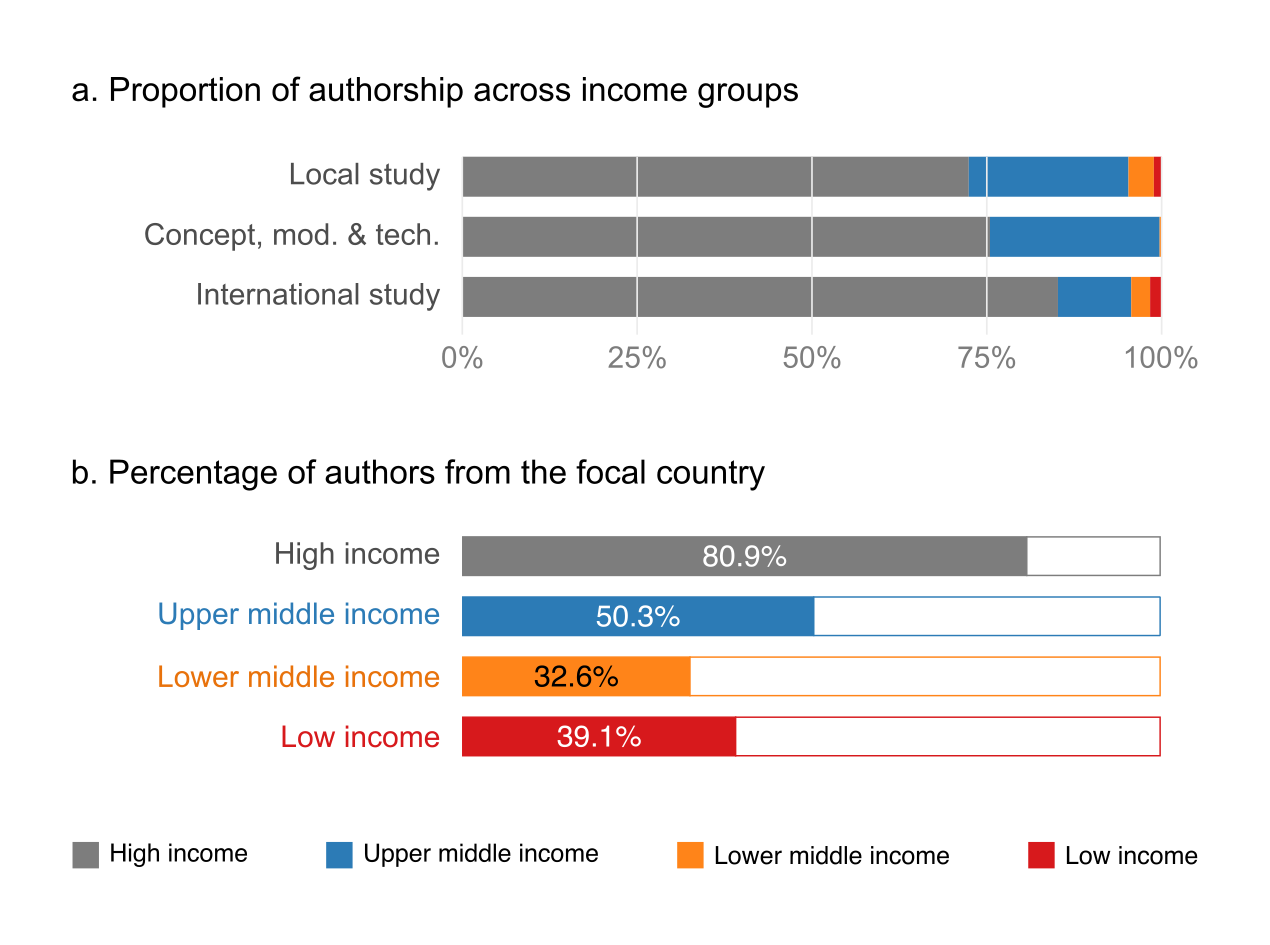

Supplement: S1 Data — (ZIP) [file pone.0273083.s004.zip › Fig4_R1_design_LLMIC_natsust_v2.png]

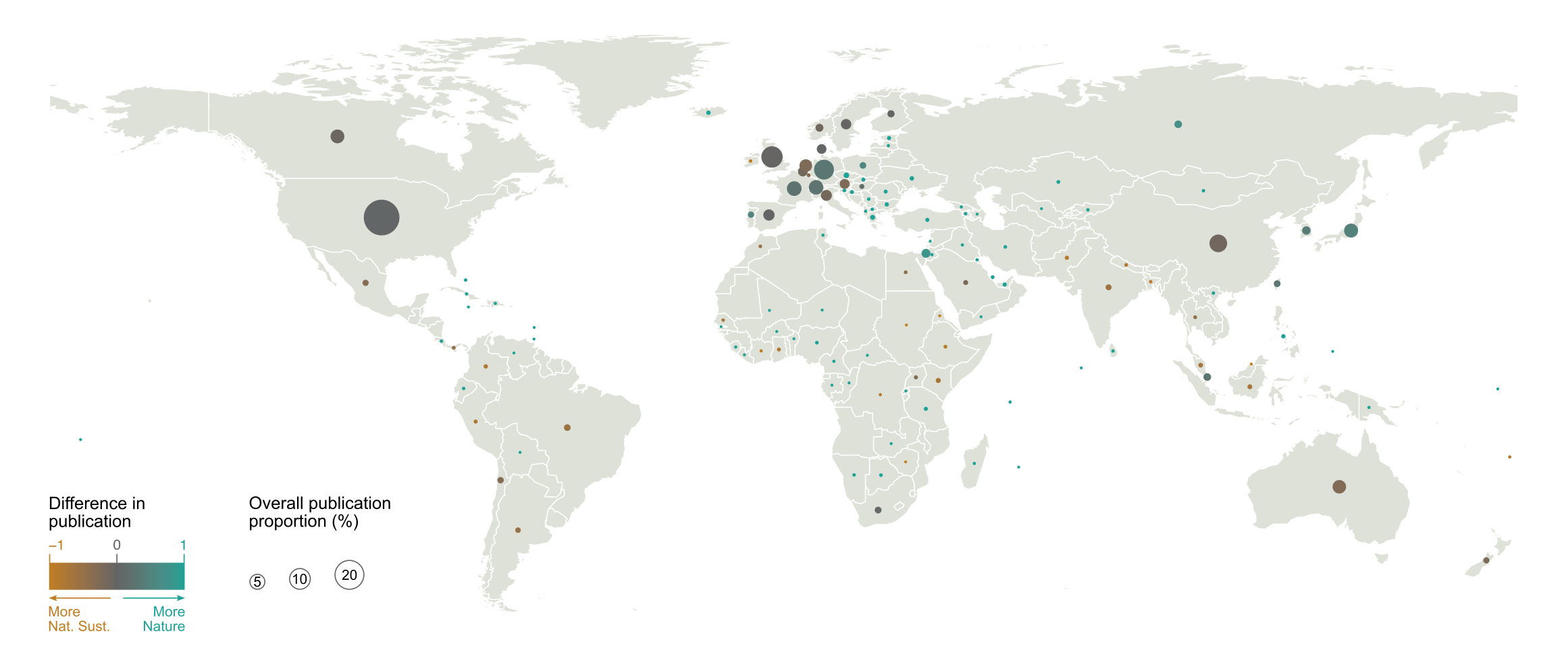

Supplement: S1 Data — (ZIP) [file pone.0273083.s004.zip › Fig3_R1_map_nat_nat_corrected_area0_v2.png]
