# Supplementary material for: Insufficient yet improving involvement of the global south in top sustainability science publications
Source: PLoS One. 2022 Sep 1;17(9):e0273083. doi: 10.1371/journal.pone.0273083 (PMC9436092; doi:10.1371/journal.pone.0273083)

S2 Figure: Co-authorship of LLMIC authors in *Nature Sustainability Local study* publications (2018-2021). *Local study* articles are those carried out in one specific country (see Material and Methods).

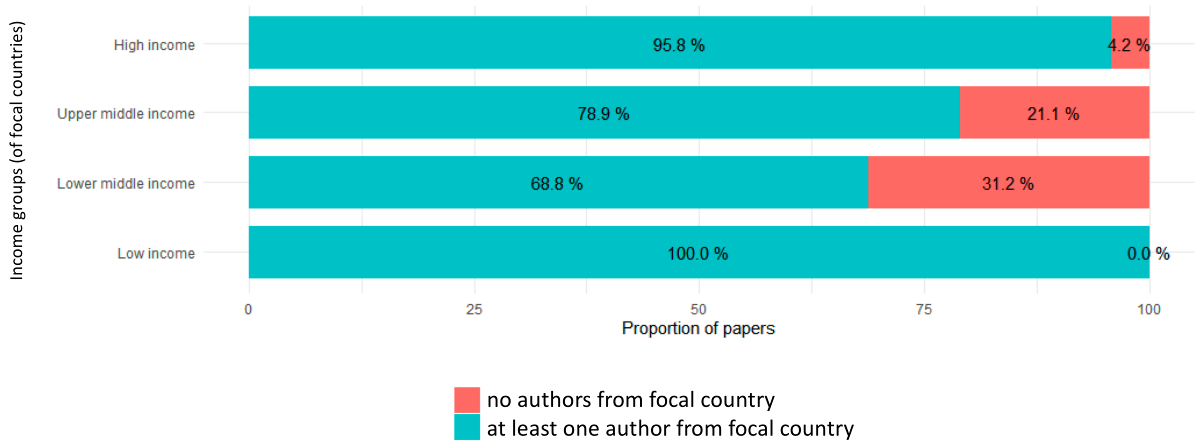

Supplement: S2 Fig — (PDF) [file pone.0273083.s002.pdf]
