# Supplementary material for: Insufficient yet improving involvement of the global south in top sustainability science publications
Source: PLoS One. 2022 Sep 1;17(9):e0273083. doi: 10.1371/journal.pone.0273083 (PMC9436092; doi:10.1371/journal.pone.0273083)
Supplement: Attachment — Submitted filename: Response_to_reviewers.docx [file pone.0273083.s005.docx]

RESPONSE TO REVIEWERS

**General comments**

1. Please ensure that your manuscript meets PLOS ONE's style requirements, including those for file naming.

Done. We have formatted the headings, figure legends, references, and author affiliations.

2. We note that you have indicated that data from this study are available upon request. PLOS only allows data to be available upon request if there are legal or ethical restrictions on sharing data publicly.

Raw data from Nature and Nature Sustainability are protected behind a pay wall. However, we are happy to share the data that have been used to build the figure and analyses. These data are now available in the Supporting information file S4. The Data Availability statement has been modified accordingly.

3. We note that Figure 1 in your submission contains map images which may be copyrighted. We require you to either (1) present written permission from the copyright holder to publish these figures specifically under the CC BY 4.0 license, or (2) remove the figures from your submission:

Data to draw the map were obtained from Natural Earth under public domain license (<https://www.naturalearthdata.com/about/terms-of-use/>). No permission is needed to use these data. The following text was added in the legend of Fig 1 and Fig 3.

The map was reprinted from https://www.naturalearthdata.com under a public domain license.

**Review Comments to the Author

Reviewer #1:**

This article discusses a very important issue about involvement of Global South in top sustainability science publications. The materials and methods section is very well written. However, it is lacking in the data analysis part. I have the following comments in particular.

1. Line number 127: When the authors claim that the entire regions of French-speaking Africa appeared totally disconnected, they must support this claim with proper arguments and bring out the reasons for that disconnection.

Right. The following sentence was added. “In *Nature Sustainability*, only 1.36% of co-authorship involved a French-speaking African country (Democratic Republic of Congo, Ivory Coast and Senegal). This corresponds to 0.04% and 0.6% of all publications in *Nature* (Ivory Coast and Senegal) and *Nature Sustainability* (Democratic Republic of Congo, Ivory Coast and Senegal), respectively.” L222-225.

2. Line number 157 to 159: In 68.8 – 100% study local researchers are involved, while 33-39% are given authorship. Does that mean the local researchers are not given authorship? If yes, what are the main reasons.

Sorry, this sentence was not clear and there was a misunderstanding here (see also our response to reviewer 3, 2j)

We have changed the sentence to: “In terms of the 28 Local study publications conducted in LLMICs, between 68.8% (Lower Medium Income, LMI) and 100% (lower income, LI) involved at least one local researcher (S1 Fig). However these local researchers represented only 33% (LMI) and 39% (LI) of the 827 authors (Fig 2b).” L248-252.

3. Discussion section: Most of the results do not follow from the study. The discussion part is a combination of literature only where the authors have cited many references. Therefore, the findings do not seem new and the reader wonders how this study is different. The authors are encouraged to do some more data analysis to distinguish their study from other studies. What is new that their study is contributing to the available literature is not very clear in the present manuscript.

We agree. We have made two text additions in the discussion:

- We have better presented why the study is new by adding the following text: “Transnational scientific knowledge that provides equal opportunities for every researcher represents a powerful way of moving towards sustainable development. In view of the UN 2030 Agenda, one would expect that sustainability science journals should act as path leaders in term of ethical partnership. However, there is no hard data in the literature about this assumption. By comparing authorship data between the journals Nature sustainability and Nature, our study intends to fill this gap.” (L283-288)
- We stick more to our results and compare with other studies (even though these comparison are limited due to methodological differences among studies): “By way of comparison, only 30% of articles in high-impact geoscience journals on an African topic contain an African author [6], while 60% of publications in coral reef biodiversity research performed in Indonesia and Philippines included host-nation scientists [5].” (L317-319)

**Reviewer #2:**

1) The paper concept is great and excellent contribution to existing body of knowledge

Thank you

2) On aspect of data and it's presentation
i) Role of article type-good to examine article (Article, Analysis and Brief communication) across regions, or across gender

As mentioned in the Methods (L 134-137), we preferred to classify the articles depending on their geographical implementation (local vs. global; field vs. Concept, modeling and technology). It made more sense for the purpose of this study than keeping the article type classification.

ii) Statistical analysis need to be improved ....any significant differences between the two journals studied?

We reviewed similar studies in the literature and did not find any robust statistical analyses to apply to our data. Our data do not allow comparing distribution but percentages, which has a limited interest.

3) i) Introduction-this is well written. On line 27, 'sister journal Nature'...can we avoid the term 'sister' (who described it a sister)?

OK; the word “sister” was removed.

ii) Line 66....'examined this issue'....would be more appropriate to state the issue, to avoid word such as this issue. Paragraph 66-79 can be re-written to improve the flow.

Good point; the two first sentences of the paragraph were re-written:

“This study examined whether ethical co-authoring is promoted in Sustainability science articles through a quantitative analysis of the 300 articles published from the first issue of Nature Sustainability in January 2018 to February 2021. We used the journal Nature as a control to test whether research published under the banner of sustainability science favored a more equitable involvement of authors from countries belonging to different income categories.” Lines 99-103.

3) Methodology
i) On materials and method section, key focus is on 'Nature Sustainability'. Nothing mentioned on Journal Nature.

Methods for the journal Nature were specified in the original MS. The reviewer may have missed them:

“For comparative purposes, we also examined publications in Nature for the same period by retrieving all research articles in HTML format from the Nature website (https://www.nature.com/) through full access (N = 2994 articles).” Lines 90-93 in the original MS.

“The title, abstract, DOI, authors’ affiliations, author contribution statements and funding information were automatically extracted from the HTML files using the ‘rvest’ R package for both Nature and Nature Sustainability publications.” Lines 94-96 in the original MS.

“For Nature Sustainability LLMIC authors (N = 80), we determined the gender of each author based on their names, while the ‘genderizeR’ R package was used for LLMIC authors from Nature (N = 384).” Lines 108-110 in the original MS.

ii) This need to be well elaborated a) Any other researcher working on such a study? What protocol did they use?

There are a great number of existing protocols for literature reviews. In our case, and as stated in the first sentence of the Methods, we performed a systematic review of all articles in two journals so that we identified all empirical evidence to test our hypothesis. Systematic methods minimize bias, thus providing reliable findings from which conclusions can be drawn.

iii) Provide a brief description of the terms; Article, Analysis and Brief communication, either on introduction or methodology so they are clear to reader.

OK; done as follows: Article (a complex story often involving several techniques or approaches, N = 185), Analysis (a new analysis of existing data or new data obtained in a comparative analysis, N = 98) and Brief communication (a concise study with up to 1,500 words, N = 17). L118-132

4) Results
Section can be improved to provide more information to readers by designing the section into various sub-themes (sub-topics) eg i) gender effect....ii) Financing....iii) article type.. etc

The analysis was divided into sub-themes in the original version of the MS: Gender (L. 1097, L150), funding (L95) and article categories (see our answer to point 2i; L85-89, Fig 2) – all line and figure numbers from the original MS.

5) Results can be improved. Line 118, use of words like; ' in that journal' should be avoided. May confuse readers as to which journal the term is referring to.

OK; changed to “in this last journal” to avoid confusion; L185.

6) On language used; This can be highly improved especially to avoid use of words such as; we (appearing severally especially on methods section),

We removed several “we” in the Methods (L117, L154, L164)

This study examined this issue (line 66),

Changed to “This study examined whether ethical co-authoring is promoted in sustainability science articles through…” (L99-100)

Yet to date evidence is lacking (line 63...reads like a continuation of previous line)

Changed to “However, to date evidence is lacking…” (L. 82)

Note that a professional English editor has revised the language.

**Reviewer #3:**

1. Very interesting and very important, if not a little limited in scope (2 journal comparison). However, the most glaring question is why not publish it in Nature Sustainability? It feels a little backhanded to publish about one journal in another. It would be interesting to know if the authors did submit it and have it rejected, and if so why? THAT would be an interesting part of the story. Asking Plos to publish this about another journal also creates a bit of an awkward friction between the two: is this going to start a heated exchange between the two? I would recommend not publishing this work in plos until it has been submitted and rejected by NS. That way, at least the process is clear.

The manuscript was initially submitted to Nature Sustainability but the editorial decision was to reject it without review. Below is a screen capture of the email:

2. Some further minor comments:

2a) L25: not clear which journal “this” refers to

Good point; we changed to “these journals”, Line 39

2b) L26: I don’t agree with the formulation that “sustainability research” favoured equity--- it’s quite a logical leap. Frame this concept more concretely

Right, our text was not clear. It was changed to “Focusing on these journals allowed us to test whether research published under the banner of sustainability science favoured a more equitable involvement of authors from countries belonging to different income categories, by using the journal Nature as a control” (L. 39-41).

Also a better framing is given earlier “Sustainability science promotes the coproduction of inter- and transdisciplinary knowledge, with the expectation that studies will be carried out through groups and truly collaborative networks.” (L. 32-34).

2c) L66: what does “this” in this issue refer to? What qualitative analysis was done? I see no evidence

The phrase was changed to “This study examined whether ethical co-authoring is promoted in sustainability science articles…” (L99-100).

By “qualitative analyses” we meant analyses that were not performed automatically extracted from the files but needed a “manual extraction (for example concerning funding). But we agree that these cannot be properly called “qualitative analyses”. This term was removed (L37, L101)

2d) L70: explain more fully and deeply this hypothesis: it’s the foundation of the paper and deserve more attention.

The hypothesis is based on the text presented earlier in the Introduction (L. 54-60 in the original MS). We have added the following sentence to better explain the more specific hypothesis about the comparison between both journals: “Indeed, as the coproduction of inter- and transdisciplinary knowledge should be carried out through groups and truly collaborative networks, we expected that sustainability research put more emphasis on the equitable cooperation among LIC-LLMIC researchers than what is currently performed in “mainstream research” (i.e. as published in Nature).” L. 106-109.

2e) L98: discuss more fully the potential impacts of considering the first affiliation only and also your decision to do this. I don’t believe that the first affiliation is necessarily representative of anything but could be simply the order in which the affiliations were entered on the platform, or simply alphabetical.

Good point. This issue is now discussed in the Methods.

Multiple institutional affiliations are a central concern in publication ethics (Bachelet et al. 2019). There are both ‘legitimate’ multiple affiliations (i.e., where institutions substantially supported the study), and ‘non-legitimate’ multiple affiliations (where at least one of the affiliations is not reflecting a substantial contribution). We assumed that by selecting the first affiliation we minimized the risk of considering a non-legitimate affiliation.

Bachelet V C, Uribe F A, Díaz R A, Vergara A F, Bravo-Córdova F, Carrasco V A et al. Author misrepresentation of institutional affiliations: protocol for an exploratory case study. BMJ open. 2019, 9(2), e023983.

Lines 145-149 and reference nº13.

2f) L105: above you say there was no co-affiliations included, so now I’m confused

Indeed, this was confusing. This is the first affiliation only so that we remove the words "or co-affiliation" in parentheses.

2g) L109: how do you know what the gender of the name is?? Malawian names, for example, are often unisex: Chimwemwe can be a man or a woman. Even more Anglo-Saxon names like Leslie can be unisex. You just can’t know!!

Good point; the Methods were not clear enough on this issue. We added the following text:

For Nature Sustainability LLMIC authors (N = 80), we determined the gender of each author based on their name, completed with Internet searches of authors’ profile with a photo. For LLMIC authors in Nature (N = 384), 29 had abbreviations as their first name and were excluded from the analysis. The remaining 355 authors were run through the ‘genderizeR’ R package (Wais 2016). GenderizeR provides a probability associated with the gender determined for a given name. We used only authors whose gender was determined with a probability > 0.75 (N = 303).

Wais, K. (2016). Gender prediction methods based on first names with genderizeR. R J., 8(1), 17.

Lines 158-164 and reference nº14.

2h) L116: are these 9 from the total or from the LLMIC numbers?

It is 9 from the 80 LLMIC (hence the percentage indicated in the text: 11.2%)

2i) L137: ratio of authors from where to where? Clarify

Right this was not clear. The lines correspond to a number of articles in co-authorship between the two countries (note that the same article is repeated "in" several lines if more than two countries are involved in the article). Circles represent the number of authors in each country. The figures have been thoroughly revised so that this problem has been fixed.

2j) L158: I don’t understand how it can be 68-100%: are these min and max values? Confusing. L159: what do “these” refer to; again, 33-39% refers to what?

Right the whole sentence was not clear. We have changed to, “In terms of the 28 Local study publications conducted in LLMICs, between 68.8% (lower medium income country, LMIC) and 100% (lower income country, LIC) involved at least one local researcher (S1 Fig), although these local researchers represented only 33% (LMIC) and 39% (LIC) of the 827 authors (Fig 2b).”. L248-252.

2k) L160: “these” what?

Right, corrected: “these local studies” L. 252

2l) L178: I would push the root cause argument back further to colonial history: it sounds like you’re blaming the poor countries. The root causes are well-documented!

Good point. The reference to the colonial history is given in the text with a reference.

“While these causes have roots in LLMICs, in some cases back to their colonial history (Gaillard 1994),…”

Gaillard J F. North-South research partnership: is collaboration possible between unequal partners?. Knowledge and policy, 1994, 7(2), 31-63.

L. 294 and reference nº16

2m) L181: yes and no: journals can’t do much except reject papers that don’t meet certain criteria, but it is really the researchers and the funders who are to blame. More details on the concrete actions if this is your main issue, but I would like to see more people sharing the blame.

We agree. The following sentence has been added:

Over the last fifty years, North-South research collaborations has moved from HICs’ researchers and funders assisting developing countries to find quick solutions to development issues to the building of local capacities in science and technology to formulating research partnership principles based on building mutual trust, learning and ownership [3]. However, in practice, these requirements are often not met for research and development projects. (L296-301)

2n) L186: again, this range is confusing

OK; corrected as in point 2j) L. 314-315

2o) L198: provide a link/reference for this Belmont Forum; similarly with the ARISE initiative below

Done

2p) Figure 1A. The lines are too thin and too similar in colour to distinguish. Using blue on blue is really not good and the difference between orange and red is imperceptible. The dots in Europe are so covered with lines they are pointless. The legend “Number of authorship” doesn’t make sense- and is it for the dots or the lines? I really like the idea of this map but it needs to be totally redesigned and maybe rethought. Figure 1B: similarly- the colours are so weak and indistinguishable: the outline on the circles is the same as the map colour which makes it even harder to see!

This is a good point. We have redesigned the figures so that our results are presented more clearly: Figure 1 has been split into Fig 1 and 3, and we added a new polar diagram to present co-authorship among countries (now Fig. 2). The calculation of the difference in publication rate between Nature and Nature Sustainability (Fig. 3) is now explained in the Methods.

To measure whether a more ethical co-authoring was promoted in Nature sustainability when compared to Nature, we calculated a difference in publication rate (Δ_PR)_) as follows:

$$\Delta_{PR}=\frac{\frac{N_{country}}{N_{total}}-\frac{{NS}_{country}}{{NS}_{total}}}{\frac{N_{country}}{N_{total}}+\frac{{NS}_{country}}{{NS}_{total}}}$$

where N_country_ and N_total_ are the number of articles in Nature for a given country and in total, respectively; and NS_country_ and NS_total_ are the number of articles in Nature sustainability for a given country and in total, respectively. Δ_PR)_ ranges between -1 (only Nature Sustainability articles) and 1 (only Nature articles).

Lines 166-173.

Fig. 1

Fig. 2

Fig. 3
