# Supplementary material for: Insufficient yet improving involvement of the global south in top sustainability science publications
Source: PLoS One. 2022 Sep 1;17(9):e0273083. doi: 10.1371/journal.pone.0273083 (PMC9436092; doi:10.1371/journal.pone.0273083)
Supplement: Attachment — Submitted filename: resonse_reviewer_R2docx.docx [file pone.0273083.s006.docx]

RESPONSE TO REVIEWER

Reviewer #3: I still think it's important to mention IN THE MANSCRIPT, not just to the reviewers, that you submitted and were rejected from Nature Sustainability. It will be a persistent question if it is not addressed up front. Also, there is a mix of italics and non italics in the namin of the 2 journals.

As requested, we have now mentioned in the discussion on the manuscript that we submitted and were rejected from Nature Sustainability. We have also put in italics the naming of the 2 journals.

L228: " The present manuscript was initially submitted to Nature Sustainability but the editorial decision was to reject it without review. Yet, we encourage this journal to develop a set of ethical partnership guidelines to attain a more balanced representation between North and South co-authors."
